# Development and Testing of a Mobile App to Collect Social Determinants of Health Data in Cancer Settings: Interview Study

**DOI:** 10.2196/48737

**Published:** 2023-09-14

**Authors:** Natasha K Oyedele, Dina G Lansey, Calvin Chiew, Cupid Chan, Harry Quon, Lorraine T Dean

**Affiliations:** 1 Department of Mental Health Johns Hopkins Bloomberg School of Public Health Baltimore, MD United States; 2 Sidney Kimmel Comprehensive Cancer Center Johns Hopkins School of Medicine Baltimore, MD United States; 3 Pistevo Decision Herndon, VA United States; 4 Department of Radiation Oncology and Molecular Radiation Sciences Baltimore, MD United States; 5 Departments of Epidemiology and Health Policy and Management Johns Hopkins Bloomberg School of Public Health Baltimore, MD United States; 6 Department of Oncology Johns Hopkins School of Medicine Baltimore, MD United States

**Keywords:** social determinants of health, mobile apps, medical oncology, mobile phone

## Abstract

**Background:**

Social determinants of health (SDOH) such as lack of basic resources, housing, transportation, and social isolation play an important role for patients on the cancer care continuum. Health systems’ current technological solutions for identifying and managing patients’ SDOH data largely focus on information recorded in the electronic health record by providers, which is often inaccessible to patients to contribute to or modify.

**Objective:**

We developed and tested a patient-centric SDOH screening tool designed for use on patients’ personal mobile phone that preserves patient privacy and confidentiality, collects information about the unmet social needs of patients with cancer, and communicates them to the provider.

**Methods:**

We interviewed 22 patients with cancer, oncologists, and social workers associated with a US-based comprehensive cancer center to better understand how patients’ SDOH information is collected and reported. After triangulating data obtained from thematic analysis of interviews, an environmental scan, and a literature search of validated tools to collect SDOH data, we developed an SDOH screening tool mobile app and conducted a pilot study of 16 dyadic pairs of patients and cancer care team members at the same cancer center. We collected patient SDOH data using 36 survey items covering 7 SDOH domains and used validated scales and follow-up interviews to assess the app’s usability and acceptability among patients and cancer care team members.

**Results:**

Formative interviews with patients and care team members revealed that transportation, financial challenges, food insecurity, and low health literacy were common SDOH challenges and that a mobile app that collected those data, shared those data with care team members, and offered supportive resources could be useful and valuable. In the pilot study, 25% (4/16) of app-using patients reported having at least one of the abovementioned social needs; the most common social need was social isolation (7/16, 44%). Patients rated the mobile app as easy to use, accurately capturing their SDOH, and preserving their privacy but suggested that the app could be more helpful by connecting patients to actual resources. Providers reported high acceptability and usability of the app.

**Conclusions:**

Use of a brief, patient-centric, mobile app–based SDOH screening tool can effectively capture SDOH of patients with cancer for care team members in a way that preserves patient privacy and that is acceptable and usable for patients and care team members. However, only collecting SDOH information is not sufficient; usefulness can be increased by connecting patients directly to resources to address their unmet social needs.

## Introduction

### Background

A growing and important body of evidence shows that social determinants of health (SDOH) can significantly influence health care delivery and outcomes in patients with cancer [1-5]. Several SDOH factors such as lack of basic resources, housing, transportation, social isolation, and substance use play an important role in the stage of cancer at the time of diagnosis and the survival rates of patients [6]. For instance, there are well-documented disparities in the incidence and survival rates according to socioeconomic status, race, education, and census tract–level poverty rate for patients with prostate and breast carcinomas [1,6]. For patients with head and neck cancer and tobacco and alcohol exposure, disease prognosis is even poorer if those patients have low socioeconomic status [7-9]. These types of findings highlight the importance of understanding, identifying, and developing strategies to mitigate the adverse effects of social challenges on the delivery of health care services for patients with cancer.

This evolving knowledge around the influence of social determinants has been acknowledged by national organizations such as the American Cancer Society’s Cancer Control Blueprint series and the American Association for Cancer Research with the goal of accelerating cancer health equity [1]. Addressing inequities related to cancer care also represents 1 of 8 key goals in the recently announced National Cancer Plan [10]. These national advocacy organizations have highlighted that unmet social needs, especially in the areas of housing, transportation, food, and social connectedness, result in disparities across socioeconomic groups and races in terms of cancer mortality rates and outcomes [11]. Optimizing the identification and management of SDOH will not only improve persistent cancer health disparities but also contribute to the goal of reducing society’s cancer burden.

Some cancer hospitals have social work care teams that assist with managing the patient’s unmet social needs, making external referrals to local community-based organizations [12,13]. The process of screening and referral services for addressing social needs varies across clinics. In some clinics, the process involves the care team screening for unmet needs, followed by patient referral and connection to resources to improve health care outcomes [12]. In other clinics, providers typically identify a patient’s social needs during the course of care or during a visit and make a referral to social work, which connects the patient to resources. A study found that, compared with traditional medically focused transition care programs, social worker–managed programs reduced 30-day readmission by 17%, were effective, and reduced costs [14]. However, social work teams require human capital resources that are costly and may not have sufficient staffing or resources to broadly meet patients’ needs. Thus, technology-based solutions to collect information about social determinants from patients and automate a response to those needs have been increasingly explored as a potentially scalable solution.

Current technological solutions for identifying and managing patients’ SDOH data have largely focused on recording information in electronic health records (EHRs). EHRs used and maintained by health care systems typically capture and store patients’ demographics, clinical histories, diagnoses, and medications and support health information exchange across providers. However, a 2019 study across a multilevel health care system, including oncology practices, reported that SDOH were not consistently collected for patients in EHRs [15]. Social isolation, housing issues, and resource strain were mentioned in <10% of the medical records [15]. Furthermore, the SDOH data that are captured may be in structured and unstructured formats, making searching and summarizing social needs a challenge [15]. In addition, EHR assessment tools are often *provider-centric* in that they are limited to data interpretation, entry, and modification of social needs by the provider, thereby creating the risk of bias assessments notwithstanding privacy concerns with the clinic environment where these assessments are conducted. The field of SDOH screening tools is also growing rapidly, with several new companies engaging in this issue. Existing patient-facing data collection tools either collect general data from patients without a focus on SDOH or collect public or organizational SDOH data instead of directly from the patient. These are limitations that can be overcome by a patient-centered tool that collects information about the patient’s SDOH on the patient’s own personal mobile device and then communicates individual and aggregate information to the care team.

### Objective

To improve the communication of social needs and ongoing changes related to them, we conducted a pilot study within 1 cancer center to inform the development and testing of an SDOH screening tool mobile app. Our screening tool collects information about unmet social needs from patients, with the potential to guide improved care of patients with cancer, especially those with a low socioeconomic position who experience health inequities. As a supplement to EHR-based tools, our patient-centric screening tool was informed by interviews with patients and providers and designed to be accessed on a patient’s own electronic device, so that they could actively participate in the process of assessing and documenting their social needs and communicate those needs to their cancer care teams, local community-based organizations, or whomever they choose. The Carealth app allows patients to self-report their social needs, which can be viewed by the care team and used to connect patients to resources. The app has the potential to increase both the timeliness and efficiency of identifying and meeting patient social needs.

We hypothesized that the use of a patient-accessible screening tool on a mobile app that collects information about the patient’s SDOH and medical record on the patient’s own device and then communicates that information to the care team would be acceptable and feasible to patients and providers.

## Methods

### Overview

We used an exploratory, sequential, mixed methods approach to develop and pilot-test the mobile app. In the first stage, we conducted formative key informant interviews with patients with cancer, providers, and social workers associated with 1 US-based comprehensive cancer center to better understand how patients’ SDOH information is collected and reported and what features of a mobile app could streamline that communication. In the second stage, we conducted an environmental scan and literature search of validated tools to feature in the mobile app. In the third stage, we developed a mobile app based on the key informant interview data and tested the app among dyadic pairs of patients and providers or care team members.

### Ethics Approval

The study was approved by the Johns Hopkins School of Public Health institutional review board (IRB; approval number 14527).

### Part 1: Recruitment for Qualitative Interviews

#### Overview

To engage a socioeconomically diverse range of patient and caregiver key informants, we recruited patients, caregivers, and community members from patient advocacy and community advisory groups associated with the cancer center to participate. Oncologists and social work key informants were recruited using purposive sampling. The cancer center’s leaders served as interviewees and then identified oncologist interviewees. Social work leaders at the cancer center identified social workers and social work office staff to be interviewed.

#### Formative Interviews

We conducted semistructured key informant interviews to understand current practice to identify, collect, analyze, and address SDOH among patients with cancer. We conducted interviews to better understand (1) what social and supportive care needs regarding health do cancer care team members and social workers hear from patients as barriers to care, (2) how and where information about social and supportive care needs is documented, (3) to which resources patients are commonly referred to meet identified needs, and (4) how software could be used to better capture the patients’ unmet needs in a way that is helpful to patients and their care team members. We conducted 30-minute interviews via telephone and videoconference with 55% (12/22) of oncologists, 27% (6/22) of patients or caregivers, and 18% (4/22) of social workers. After obtaining oral consent, interviews were recorded and transcribed. Sample sizes for the semistructured interviews included sufficient participants until data saturation was achieved or the point when no new information was gleaned from continued data collection. Interviews were conducted between December 2021 and July 2022.

#### Analysis

Interviewers followed a semistructured interview guide with questions that focused on social needs data collection, clinical decision support for the identified social needs, and software needs for documenting the social needs. Interview questions were tailored to the role of the interviewee (eg, care team member, patient, or caregiver). We began thematic analysis during data collection using Excel (Microsoft Corporation). The initial analysis was broad and based on the interview guide domains.

We analyzed data about the common patient social need challenges in accessing cancer care and the documentation and referral for identified patient social needs. To organize and support the analyses, we developed an analytic memo that described all observed themes, including how each interviewee fit within each theme.

### Part 2: Identifying Patient-Centered and Evidence-Based SDOH Measures

We then performed an environmental scan of the SDOH instruments. We reviewed sources from PubMed for published and psychometrically valid self-administered instruments that measured an aspect of SDOH. Keywords included the following: “([cancer]) AND ([social determinants])” (with 7533 results) and “([cancer]) AND ([social needs])” (with 2806 results). For both searches, the results were sorted based on best match, and we reviewed the first 10 pages for relevant articles. Of the results, we also reviewed the related papers listed for the primary responses. In addition, we searched the PhenX Toolkit (RTI International) [16] and HealthMeasures Patient-Reported Outcomes Measurement Information System (PROMIS) [17]. Within the PhenX SDOH domain, we reviewed the 12 individual SDOH and 10 structural SDOH tools to identify any that were specifically validated or designed for use among patients with cancer. Regarding PROMIS, we used the search function to identify relevant measures, yielding 77 PROMIS social health measures, which were then further reviewed for relevance to cancer outcomes. In addition, we reviewed 5 PROMIS measures that were specific to cancer. We included measures that were validated (ie, the psychometric properties of which had been assessed and published), were cancer specific, had the ability to be completed as a self-administered survey, captured multiple SDOH, and exhibited low time burden for patients. We selected measures from key domains mentioned in the qualitative interviews that focused on food, housing, social connections, and transportation. Domains represented in the environmental scan, such as health literacy, distress owing to cancer-related changes in life, and other non–cancer-specific social needs, were also selected for inclusion.

### Part 3: Design and Pilot Study of Software for SDOH Screening Tool

#### Overview

**Figure 1:**
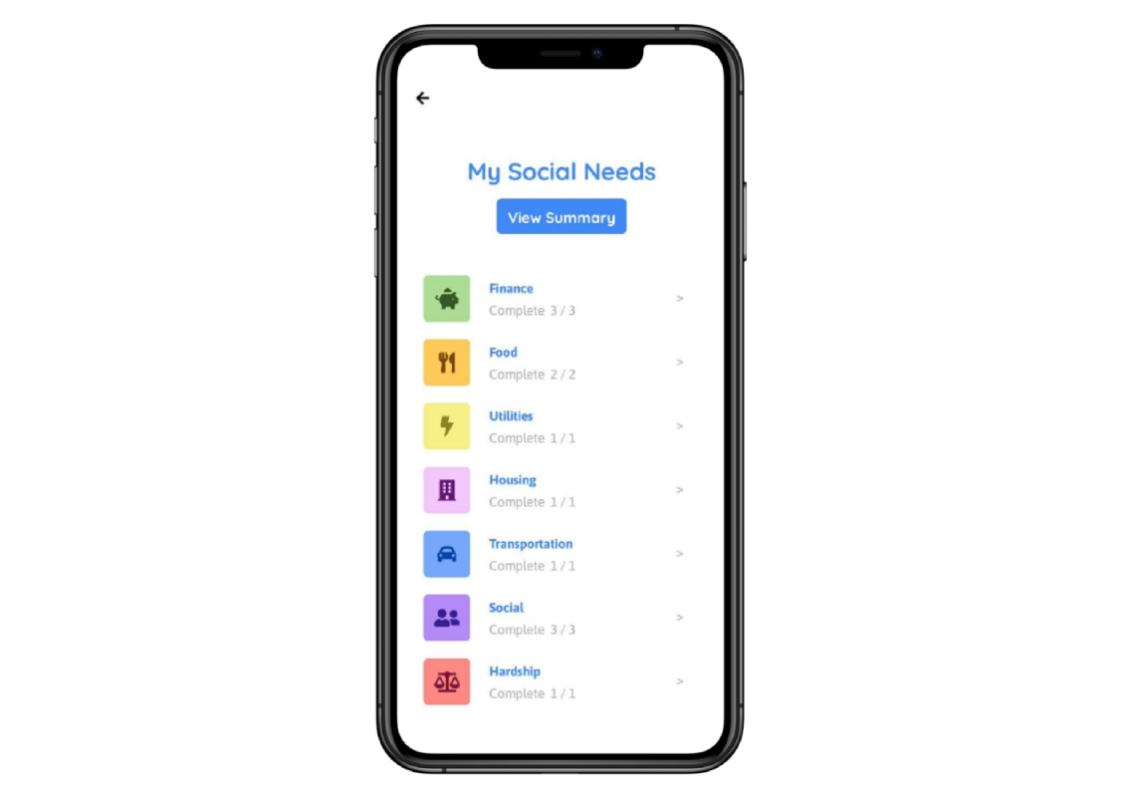
Patient-facing social determinants of health screening tool mobile app.

On the basis of SDOH domains identified through interviews and literature search findings, a total of 36 questions were chosen for the mobile app pilot data collection. Questions were selected to identify patient needs across 7 categories: finance, food, utilities, housing, transportation, social support, and financial hardship, each differentiated by color, and questions about patient demographics (Figure 1). The app’s summary page displayed the number of answered and unanswered questions to provide an overview of the patient’s social needs.

In parallel with the mobile app, a web-based platform was developed for providers to view patients’ survey responses. The provider-facing web-based application allowed the approved providers to see the patients’ survey responses. To streamline the process, the provider’s app view highlighted the important social needs that patients identified on patient input in the patient-facing app. All patient survey responses were available for provider view; responses that indicated a social need were highlighted. This allowed the provider to easily spot if there were unmet social or supportive care SDOH needs that required their attention to assist the patient.

#### Recruitment for Pilot Testing

We conducted a pilot study of the mobile app prototype with dyads of clinical providers (eg, oncologist, nurse, or nurse practitioner) and patients. To recruit providers, an overview of this study was presented during standing disease-specific clinical research group meetings, and we shared a 1-page summary of the study. To recruit social workers, we coordinated with the director of Patient and Family Services. Interested care team members (eg, providers, nurses, and social workers) volunteered to be part of the study. Verbal consent was obtained from those interested in participating in the study. After enrolling in the study, providers, nurses, and social workers identified interested patients within their clinics.

Patient eligibility criteria included the following: (1) aged ≥18 years; (2) had a diagnosis of breast, prostate, or head and neck cancers >3 months ago and determined to be clinically stable by their oncologist (clinically stable refers to patients who have cancers that are not actively progressing; this can also include patients under ongoing treatment but who have minimal side effects of their cancer treatment that affords participation in a research study); (3) scheduled for a follow-up patient visit at the comprehensive cancer center; (4) able to read and respond in English; (5) have access to a smartphone or computer with an internet connection and web browser; and (6) able to provide consent to the study.

Following the identification of an interested patient, the care team member was sent an IRB-approved email message and flyer to be forwarded to the patient’s email or EHR to make the patient aware of the study. The care team member subsequently contacted the study research assistant to alert them about the interested patient. With permission from the referring care team member, the research assistant followed up with the patient via phone and email to conduct the eligibility screening using an IRB-approved script to explain the study, answered the patient’s questions about the study, and confirmed eligibility using screening questions. The research assistant emailed the instructions about how to access and review the pilot testing consent documents to eligible patients. Patients gave verbal consent and confirmed their response via an email to the research assistant. Recruitment and enrollment for the pilot test were conducted from June 2022 through September 2022.

#### Acceptability and Feasibility Testing

Patients accessed the screening tool through the Carealth app on their personal mobile device and responded to the self-administrated, 36-question survey before their clinic visit with the provider. Providers accessed the patient’s survey responses before their visit with the patient, using a provider-accessible web-based platform. As the Carealth app did not connect patients to resources, the study team made the care team aware of the reported needs, so that they could be met through the standard-of-care process (ie, social work or provider referrals to social work). In addition to recording patients’ social needs through the app, we also used the validated Acceptability E-scale [18] to assess 6 elements of usability and acceptability: ease of use, understandability, enjoyability, helpfulness, time required, and overall satisfaction among patients and providers. Patients completed the Acceptability E-scale survey questions immediately upon completion of the survey using the Carealth app. The research assistant followed up with brief qualitative interviews for app feedback on the day of and 2 weeks after the clinic visit.

Providers responded to a questionnaire adapted from the validated System Usability Scale [19] regarding the usefulness of the app. Likert scale survey questions included the following: (1) “this app gave my patients better control over sharing social needs with the cancer care team,” (2) “this app helped me to better understand what my patient’s social needs are,” (3) “this app helped me more easily coordinate care with other members of my patient’s care team,” (4) “I prefer using this app to learn about the social needs of my patient,” (5) “I was comfortable using this app to address social needs of my patient,” and (6) “this app helped me more easily connect my patient to resources to address their social needs.”

#### Analysis

We calculated and reported the frequencies of the patient demographic characteristics including patient sex assigned at birth, Hispanic ethnicity, race, education, and household income. We reported the mean and SD for patient age, measures of frequency descriptive statistics for SDOH needs, and patient usability and acceptability metrics.

## Results

### Overview

We interviewed 22 participants, including 5 (23%) patients, 1 (5%) caregiver, 4 (18%) social workers, and 12 (55%) oncologists. Patients and care team members responded similarly to the semistructured key informant interview questions. Care team member interviews provided insight about the collection and documentation of patients’ social needs. Patient and caregiver interviews revealed similar themes of common social need challenges among patients. Given the consistency, we report findings aggregated according to interviewee subgroups.

### Formative Interviews With Patients With Cancer and Caregivers

Patients and caregivers reported challenges such as transportation to and from medical appointments, lack of financial security, food insecurity (specifically, lack of access to nutritious foods), and social isolation owing to lack of a support system. Low health literacy, which affects a patient’s ability to advocate for themselves during cancer diagnosis and treatment, was also noted as a common challenge in accessing cancer care. Patients and caregivers indicated that all members of their care team—oncologist, primary care provider, social worker, and nursing team—should have access to their social needs information entered into the app to ensure that the care team can take action as quickly as possible to direct them to resources to meet their reported social needs. Patients emphasized the need for cancer care teams to take action and direct patients to resources to meet their social needs. They emphasized the importance of communication within clinical teams and with patients. Patients wanted assurance that an action plan would be put in place to address the expressed social needs, highlighting the importance of SDOH.

When asked about what might increase a patient or caregiver’s interest in using an app that collects information about SDOH, a patient noted the importance of clinical teams acting on the social needs information shared:

If I really think someone is going to help me...then I would be happy to fill it [survey] out and talk to them.

Patient or caregiver 1

When asked about concerns that patients or caregivers might have about providing social needs information in an app to allow their clinical care team to see the data, concerns about confidentiality, privacy, and embarrassment were cited as possible reasons why patients may be hesitant to share:

I think sometimes people are embarrassed or reluctant to share, so they may want to [use the app] to share on a bigger level or be willing to share on a bigger level, as opposed to the people they see all the time...them knowing the people and embarrassed about it.

Patient or caregiver 4

There is probably a matter of a lot of pride involved. It might be difficult to share something that personal.

Patient or caregiver 5

### Formative Interviews With Cancer Care Team Members

A social worker interviewee similarly noted that transportation to the medical facility, access to food, financial insecurity, and social isolation are common challenges for patients. The financial support to address these social needs is the driving factor for patients:

While we offer free counseling and a variety of mental health related types of services, almost no one knocks on our door for that. They come seeking a concrete related need...transportation, access to medication, housing and alike. Those all have to center not around the domains of the namesake [SDOH] but around finances. The things that drive people to our door are money, and money and money.

Social worker 3

An oncologist expressed that providers see value in collecting social needs information from patients but often do not know what to do about the identified social needs:

I use the module for social determinants of health [in EHR], and it is documented right there...is there stress, do they smoke, do they have transportation, how active are they, are they isolated. It’s a nice module.

Oncologist 6

...Patients like somebody asking about social needs right then and there and it gets documented, but it doesn’t go anywhere! If I find that someone is moderately isolated, that’s for me to say “moderate isolation” what do I do with that, I have no idea. Is that a trigger for me to send to a social worker? I don’t know what that means. If they can’t get in to see me, I can figure that out...I don’t know if it [collecting the social needs information] helps me or the patient or if there’s follow-through.

Oncologist 6

Providers noted that a social needs survey-based assessment and visualization tool currently exists in EHR; however, patients do not have access to them. Providers often did not know that the tool was available, and those who could access the tool did not proactively use it. The provider sentiment was that SDOH are important, but their critical role in patient health is not emphasized owing to structural challenges:

Most of the faculty/providers don’t know it [the module in EHR] exists, don’t know to go there, they’re not required, there hasn’t been any initiative to get even 10% of people to complete. My frustration is I think it’s important and I add it and it populates into my note if I wanted it to, but I haven’t been instructed on how to use it or connection to anybody or what threshold makes it. If it’s screening, it’s screening and goes nowhere.

Oncologist 6

Doctors have limited time to see each patient...They give us 20 minutes to see each patient. In those 20 minutes, I could make mistakes that result in loss of license or malpractice. But I don’t get dinged for ignoring social determinants of health.

Oncologist 9

The findings from the qualitative interviews confirmed that the mobile app solution developed should address issues of transportation, food, finances, and social relationships after a cancer diagnosis; be accessible to both patients and providers; and offer clinical decision support once these needs were identified.

### Environmental Scan

In the first phase of the environmental scan, we searched for cancer-specific screening tools. In the second phase, we searched for SDOH screening tools more broadly and were not cancer specific. Half of the SDOH measurement instruments identified were not specific to the needs of patients with cancer, with exceptions of cancer-related toxicity, physical function, and supportive care needs. Most instruments, except for the Protocol of Responding to and Assessing Patient’s Assets, Risks, and Experiences tool and the Accountability Health Communities Model, focused on 1 specific aspect of social determinants, rather than assessing social determinants broadly. On the basis of the screening tools identified, we balanced efficiency with patient burden to select screening tools that were to be included in the app. In some cases, we used screening tools where we would get the most SDOH information in the shortest time or screening tools with a threshold to determine when a patient needed intervention. Thus, the mobile app was built to include a series of brief instruments, each of which focused on a different type of social need (ie, cancer-related financial toxicity, food, housing, transportation, and social isolation). To reduce patient burden, we selected measures from key domains mentioned in the qualitative interviews and domains represented in our environmental scan, such as health literacy and distress owing to cancer-related changes in life. Other social determinants data were captured through validated tools commonly used by health professionals, and the focus was on a general set of social needs that are not cancer specific (eg, Accountability Health Communities Model and Protocol of Responding to and Assessing Patient’s Assets, Risks, and Experiences; Table 1).

**Table 1:** Data collection tools used in the social determinants of health (SDOH) screening tool mobile app.

SDOH screening tool	Description	Study, year	SDOH domains
Protocol for Responding to and Assessing Patient Assets, Risks, and Experiences	It is a nationally standardized screening tool designed to equip health care and community partners to better understand and act on individuals’ SDOH.	Weir et al [20], 2020	Housing Finance Employment Transportation Social and emotional health Income
Accountability Health Communities Model	It is a health-related social needs assessment—a 10-question screening tool to identify unmet needs across 5 core domains.	Billioux et al [21], 2017	Housing Food insecurity Transportation Utility needs Interpersonal safety
CHLT-6^a^	It is a tool designed to identify individuals with limited cancer health literacy. CHLT-6 scoring yields the probability of belonging to the limited cancer health literacy class and the probability of belonging to the adequate cancer health literacy class.	Dumenci et al [22], 2014	Cancer-specific health literacy
National comprehensive cancer network distress thermometer	It is a screening measure to identify and address psychological distress in individuals with cancer. Respondents are asked to indicate the number (0-10) that best describes how much distress they have been experiencing in the past week, including the day of measurement.	Cutillo et al [23], 2017	Psychological distress

^a^CHLT-6: Cancer Health Literacy Test–6.

### Pilot Study

**Figure 2:**
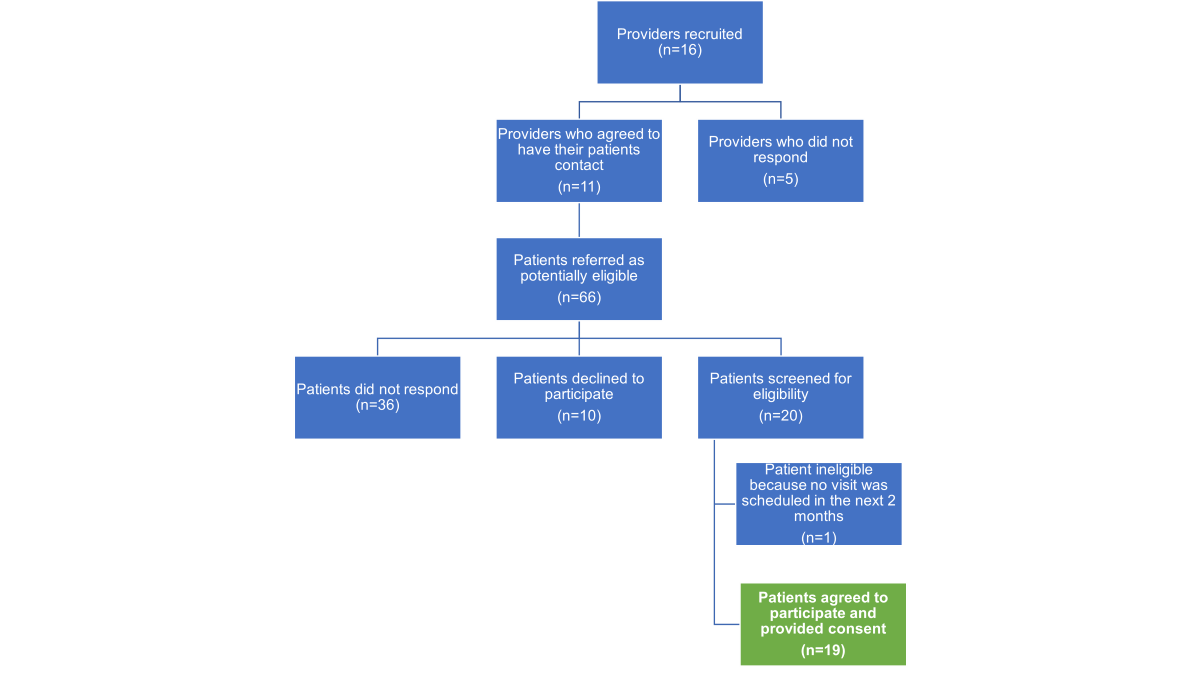
Flowchart of provider and patient pilot study recruitment.

We enrolled 11 providers who identified and referred 66 patients who were interested in our study. Among the 66 potentially eligible patients, 10 (15%) declined to participate and 36 (55%) did not respond following initial outreach. Among the 30% (20/66) of the patients screened, 5% (1/20) did not have an upcoming scheduled visit and was deemed ineligible. The remaining 95% (19/20) of the patients were eligible, and all eligible patients were enrolled. Among the 19 enrolled patients, 16 (84%) entered data into the app (Figure 2). The remaining patients were unable to complete data entry owing to disease progression, death, or hospitalization.

Characteristics of the 16 patients are summarized in Table 2. Patients who pilot-tested the app were, on average, aged 62 (SD 11) years; mostly men (10/16, 63%) or White (11/16, 69%); and had college-level education. A total of 7 (N=16, 44%) had an annual household income of ≤US $70,000, and 5 (N=16, 31%) patients met the threshold of <200% of the federal poverty line. Most patients were from head and neck cancer clinics (6/16, 38%) and prostate cancer clinics (6/16, 38%); the remaining were from breast cancer clinics (3/16, 19%), with representation from patients across most cancer stages: unstaged (5/16, 31%), stage 2 (3/16, 19%), stage 3 (2/16, 13%), and stage 4 (6/16, 38%).

Overall, one-fourth (4/16, 25%) of the patients in the pilot sample reported needs for instrumental help with basic requirements such as food, utilities, housing, or transportation to medical visits, with a few freely responding that their needs included “clothing,” “food,” or “medicine or any healthcare.” Overall, cancer distress was low (mean 2 [SE 2], of a possible range of 0-10, with 10 indicating high distress and 3 indicating the recommended cutoff), with 13% (2/16) of the patients indicating substantial distress. Just under half of the patients (5/16, 31%) had challenges with loneliness or social isolation (Table 3).

**Table 2:** Characteristics of patients who provided data for the mobile app pilot study (n=16).

Demographic characteristics			Values
Age (years), mean (SD); range			62 (11); 35-82
**Sex (assigned at birth), n (%)**			
	Female	6 (38)	
	Male	10 (63)	
Hispanic, n (%)			0 (0)
**Race, n (%)**			
	Asian	1 (6)	
	Black	4 (25)	
	White	11 (69)	
**Education, n (%)**			
	High school (9-12 y)	4 (25)	
	College (13-16 y)	7 (44)	
	Higher than graduate school	5 (31)	
**Annual household income (US $), n (%)**			
	<10,000	2 (13)	
	10,000-30,000	3 (19)	
	30,001-70,000	4 (25)	
	>70,000	7 (44)	

**Table 3:** Reported social and economic needs among patients who provided data for the mobile app pilot study (n=16).

			Values
**Any social need^a^, n (%)**			4 (25)
	Food	1 (6)	
	Utilities	0 (0)	
	Housing	3 (19)	
	Transportation	2 (13)	
Social interactions <5 times/wk, n (%)			7 (44)
Activities of daily living, n (%)			0 (0)
Loneliness, n (%)			5 (31)
Cancer distress, mean (SE); range			2 (2); 1-5

^a^Respondents could endorse >1 social need; percentage values may not sum to 25%.

### Patient and Provider Impact Metrics

**Figure 3:**
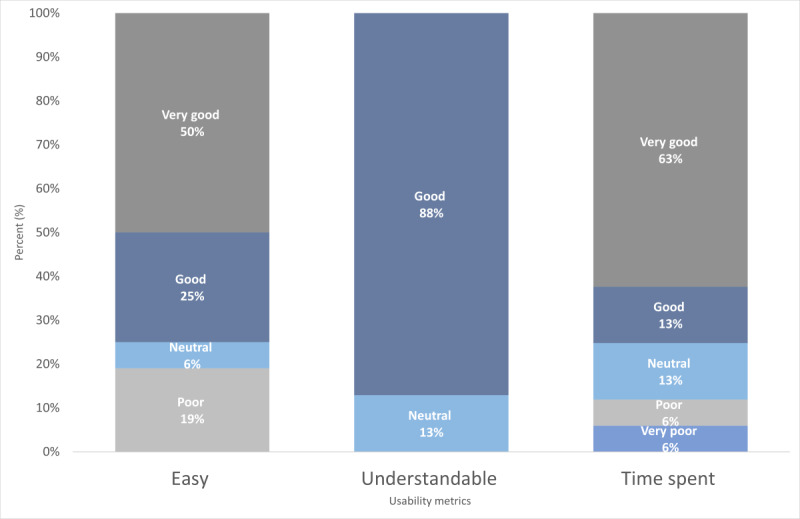
Usability metrics of patients in the pilot study.

**Figure 4:**
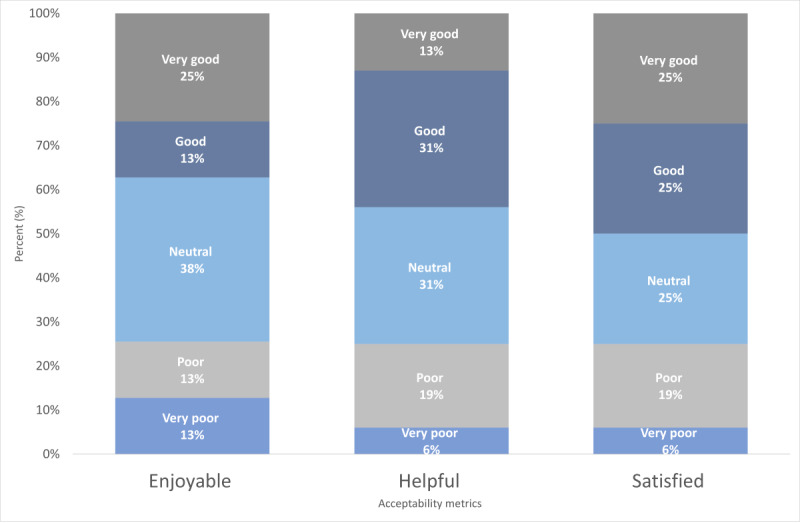
Acceptability metrics of patients in the pilot study.

Patients rated the app high on usability, with scope for improvement on acceptability. Overall, patients were most likely to rate the app as easy to use, with 75% (12/16) reporting good or better; understandable, with 88% (14/16) reporting good or better; and taking a reasonable amount of time, with 76% (12/16) reporting good or better (Figure 3). Reports of enjoyability and helpfulness saw great spread, with approximately one-fourth of the patients (4/16, 25%) rating enjoyability and helpfulness as poor or very poor. Half of the patients (8/16, 50%) gave the app high ratings regarding satisfaction (Figure 4). Some of the low ratings of enjoyability, helpfulness, and satisfaction were driven by 13% (2/16) of the patients who indicated a social need and reported that they were less likely to find the app helpful or enjoyable.

Patients found the app as easy to use and thought it allowed for more private disclosure of needs. In the follow-up interviews with patients, a patient noted the following:

The app was easy to use once I was able to log-in. The log-in process may be difficult for a less technologically savvy person.

Overall, 29% (4/14) of the interviewees had difficulty during the log-in process, but once the issue was resolved, patients reported that the app and survey completion were straightforward:

The app allows for greater privacy, rather than disclosing my social needs during a visit.

Patients found the app enjoyable and satisfying to use but noted that the app could have been more helpful. This may be because the phase-1 pilot study was focused on usability and acceptability of the Carealth app and did not link patients to resources to address social needs reported in the app. Patients found that the app captured valuable information. Overall, 86% (12/14) of the interviewees noted that the survey accurately captured the social needs that influence their care:

...The app accurately captured my needs, and was thorough covering food and transportation.

Although some patients reported the app not being helpful in the quantitative data collection, only 7% (1/14) of the patients mentioned this in the interview, even after probing. This patient was a patient with metastatic cancer who felt that it did not capture the dynamic nature of changes in the life of someone with metastatic cancer. Other patients mentioned that the app was the most helpful when it was attached to meeting a specific need:

I would use the app for a more direct search of social needs resources.

The app asked all the right questions, and I would be able to get the help and resources needed.

Providers strongly endorsed the app’s value. A subset of providers also shared their perceptions about the app, finding it overall helpful and endorsing the app. There were 6 responses, all of whom were from nurse practitioners, representing provider feedback regarding the mobile app based on a set of custom questions about the usefulness of the app. All providers (6/6, 100%) responded “agree” or “strongly agree” to questions related to their perceptions about the Carealth app.

### Patient Follow-Up Interview After 2 Weeks

Follow-up qualitative interviews were conducted with patients after completing their clinic visit. There were 14 (74%) responses from the 19 patients enrolled, representing feedback about the mobile app; 16% (3/19) were unreachable owing to developing health challenges (eg, cancer recurrence), whereas others had personal schedule changes that made them unreachable. Responses were based on a set of qualitative interview questions about the use of the app following their clinic visit and follow-up based on the social needs information shared. Of the 14 patients, 5 (36%; n=1, 7% head and neck cancer; n=2, 14% breast cancer; and n=2, 14% prostate cancer) used the app following their clinic visit. Among the 5 patients, those with breast cancer shared the social needs summary report with their social worker and peer navigator and received follow-up resources relevant to their social needs. Overall, 14% (2/14) of the patients intend to share the summary with their primary care provider at a future visit and 14% (2/14) of the patients stated that they might share the summary report with other providers at future visits.

## Discussion

### Principal Findings

We developed and pilot-tested a mobile app for actively collecting SDOH data to identify social needs among patients with cancer. Our objective was to improve communication of social needs and ongoing changes related to them and assess the usability and acceptability of the Carealth mobile app. Patients reported that the mobile app was easy to use, accurately captured their social needs, and gave them the desired privacy when sharing sensitive social needs. The Carealth app does not require the patient to acknowledge their social needs out loud to their provider with the potential of being overheard during the clinic visit or inadvertently seen by onlookers if screening is conducted in some other public space (eg, a clinic waiting room). However, the app had scope for improvement on being enjoyable or helpful. Patients expressed the desire for the app to direct users to resources that could address the identified social needs. This emphasizes that it is not enough to simply collect data about patients’ needs but that patients need follow-up support and resources to have their needs met. Some of the low and neutral ratings of enjoyability, helpfulness, and satisfaction were driven by 13% (2/16) of the patients who represent half of those indicating a social need, and they were less likely to find the app helpful or enjoyable. We attribute this to the fact that the app was part of a pilot study that was only designed to demonstrate that we could collect information about social needs but not designed to address the social need. Findings about feasibility and low to moderate acceptability suggest that the app is already perceived as highly usable to patients and even more so to providers.

In previous studies and in our study, acceptability of SDOH screening among patients with cancer is high. Patients found electronic, cancer-specific, social risk factor screening to inform cancer care to be acceptable in a pilot survey at 2 Philadelphia cancer centers [24]. Patients also found supportive care screening to be highly acceptable when incorporated into routine cancer care [25]. Patients with cancer found a validated questionnaire developed to assess unmet supportive care needs as an acceptable tool and supported in-person screening for patients with cancer [26]. However, few studies have made direct comparisons of the modes of SDOH screening administration, comparing in-person screening versus screening using a mobile app. Our study also did not make a comparison of SDOH screening between the 2 modes but, given that in-person SDOH collection through patients sharing with providers is the standard of care, our results found that patients prefer the privacy that the app provides.

Our findings demonstrate the utility of a mobile app that allows patients and providers access to social needs information. Uniquely, our mobile app advances provider and health systems collecting data about SDOH to offer a patient-centric platform for sharing and updating SDOH information longitudinally. To manage the influence of unmet social or supportive care SDOH needs, health care institutions need to measure and address their influence. This approach represents an important strategic and wide-reaching goal for the cancer community. Our pilot study demonstrated that a mobile app could be a potential tool for patients and providers to communicate about a patient’s unmet social needs, while also preserving patient privacy.

Given the complexity of cancer care, the potential variation of social needs, which can be dependent upon cancer diagnosis, stage of disease, disease status, treatment plan, and desire to efficiently streamline the collection and reporting of SDOH challenges that patients face, our study expands on previous studies in primary care clinics documenting the infrastructure, software, and SDOH screening tools used in oncology clinics. Our mobile app demonstrates the feasibility of screening for social needs with patient input, with the potential for resource referral to address unmet needs. Although an EHR-based approach allows providers and care teams to identify and document patient social needs, SDOH data are summarized in formats that make the provider’s ability to search and summarize social needs a challenge and restrict patient viewing. Our app gives patients a summary of their social needs and allows for sharing and updating SDOH information. It allows providers to access a summary of patients’ self-reported social needs to easily identify important and unmet patient social needs that require attention. The opportunity for 2-way communication about ongoing changes in their unmet social needs engages the patient in discussing the social needs vital to their cancer care.

Our social needs app demonstrated face validity as an acceptable solution for documenting the unmet social needs of patients with cancer in a way that is accessible to patients and care teams in the health care system where they receive care. The low ratings of helpfulness, especially among patients with social needs, further suggest that patients prefer solutions that refer them directly to social needs resources. Although our app did not link social needs data to resources to meet needs or clinical outcomes, it demonstrated the feasibility of an app to accurately capture data while maintaining the patient’s privacy. To demonstrate full efficacy, future trials of a patient-centered mobile app could be more helpful to both patients and providers by connecting patients to resources to address their reported social or supportive care SDOH needs and provide clinical decision support to guide members of their cancer teams about how best to address patient-reported social or supportive care SDOH needs.

### Limitations

Our study was limited in sample size, and patients were only enrolled from 1 cancer center. This limits the ability to conduct regression-based hypothesis testing and limits our ability to generalize beyond our cancer center. Despite this limitation, our study had patients with cancers across multiple tumor sites, representing highly prevalent cancers and patient populations with low socioeconomic position. For the app’s pilot testing, no concrete health outcomes were assessed; this was a pilot study to assess the usability and acceptability of the app and was not intended to assess health outcomes. Less than half of the patients (5/16, 31%) who participated in the app’s pilot testing identified as Asian or Black. The demographics of the final sample reflect the study attrition and patients who completed the follow-up visits. This limits our ability to generalize the results to a more diverse sample. Finally, this was not a comparability study; therefore, we cannot compare our findings with existing EHR solutions; however, our qualitative interviews and acceptability ratings suggested that providers thought that the patient-centric app might be better for visibility to patients and providers.

### Conclusions

Our patient-centric SDOH screening tool overcomes the limitations of EHR-based screening and management tools by integrating the patient’s input in the design and collection of data. Patients and providers found the mobile app solution to be acceptable and feasible and that it protects patient privacy, yet felt that it had scope for improvement on being helpful. Future development goals of an SDOH app should link key oncologic delivery measures and outcomes that would drive the decision support recommendations and referrals to the clinical care team for use in a clinical setting and support the communication of SDOH information to the clinical care team directly and to community-based resource providers. Finding an interface between the Carealth app and the existing EHR-based screening tool is a worthy area of future development.

## Abbreviations

EHR: electronic health record
IRB: institutional review board
PROMIS: Patient-Reported Outcomes Measurement Information System
SDOH: social determinants of health
